# 3D Printing Parameter Optimization Using Taguchi Approach to Examine Acrylonitrile Styrene Acrylate (ASA) Mechanical Properties

**DOI:** 10.3390/polym14163256

**Published:** 2022-08-10

**Authors:** Abdul Zubar Hameed, Sakthivel Aravind Raj, Jayakrishna Kandasamy, Muhammad Atif Shahzad, Majed Abubakr Baghdadi

**Affiliations:** 1 Department of Industrial Engineering, Faculty of Engineering, King Abdulaziz University, Jeddha 21589, Saudi Arabia; 2 Department of Manufacturing Engineering, School of Mechanical Engineering, Vellore Institute of Technology, Vellore 632014, Tamil Nadu, India

**Keywords:** ASA polymer, fused deposition modelling, infill density, L18 orthogonal array, 3D printing

## Abstract

Polymer composites with different reinforcements have many applications. By adjusting process settings and adding fibers and fillers, composite properties can be improved. Additive manufacturing is popular in the polymer industry because it can manufacture intricately designed parts with fewer defects and greater strength with less material consumption. Composites use thermoplastics and thermosetting polymers. Thermoset plastics cannot be reused or recycled; therefore, they are disposed in landfills, creating pollution and environmental harm. In this work, thermoplastic ASA (Acrylonitrile Styrene Acrylate) polymer filament is used for FDM 3D printing. The specimens are made by varying five process parameters that affected the materials’ mechanical properties. The tensile, flexural and impact specimens are made using MINITAB software and ASTM requirements. The L18 orthogonal array experimental design, specimens and results were optimized. Infill density and layer height were most influential. Maximum tensile strength of 51.86 MPa, flexural strength of 82.56 MPa and impact strength of 0.180 J/mm^2^ were obtained by following the software-suggested input factors and compared with the predicted values. Final error percentage was obtained between the predicted and the experimental results and it was found to be under 3%, which is acceptable.

## 1. Introduction

As early as the Paleolithic period, humans were able to produce things for their everyday needs; they had a material sense and used it to create tools for their own gain. However, the manufacturing process for the tools was based on subtractive techniques, in which the original components are turned into useful goods by removing surplus bulk material until the final object is achieved. The subtractive method of production has progressed substantially and new techniques continue to emerge. In the early 1980s, a paradigm known as additive manufacturing was established. It has proven to be an excellent technique for creating any geometry [1].

In the FDM process, different thermoplastic materials are utilized to print different complex shaped objects. Different materials used in the FDM process include Polylactic acid, PLA-based graphene, Acrylonitrile butadiene styrene, Polycarbonate, NinjaFlex 85 A, Polycarbonate–ABS, Polyurethane elastomer, thermoplastic Polyurethane and Carbon-Silicone rubber [2]. PLA is derived from renewable resources and is compostable. It will not produce toxic fumes during printing or when incinerated. It is basically derived from crops. ABS is usually used for processing foods and has improved resistance against impact, abrasion and chemicals. It has better machinability, it can be thermoformed and it is flexible enough to print complex shaped jobs. It has better electrical properties and dimensional stability also. Polycarbonate has better thermal insulation when compared to other thermoplastic polymers and is light in weight. It has UV protection properties. NinjaFlex 85A is used in making sporting goods, in healthcare and in industrial manufacturing. It features good flexibility, excellent elongation properties, low friction and abrasion resistance. Thermoplastic Polyurethane has good load bearing capability, resistance to oil and ozone, and has good abrasion resistance also. Very few materials are used in making 3D-printed membranes, and for printing the membranes only a few additive manufacturing processes are used. Processes like electrospinning, sintering, stretching, track etching and phase inversion are used in printing membranes [3].

3D printing has introduced a new approach for layer-by-layer creation of items from the bottom up. By adding successive layers of material in response to computer-controlled instructions, additive manufacturing makes parts. People have grasped the significance and benefits of additive manufacturing as its popularity has increased relative to conventional techniques. Compared to conventional or subtractive manufacturing techniques, additive manufacturing has progressed and produced more promising results. It can be employed in small-scale production for customization and personalization, whereas conventional methods are limited to mass production.

**Figure 1:**
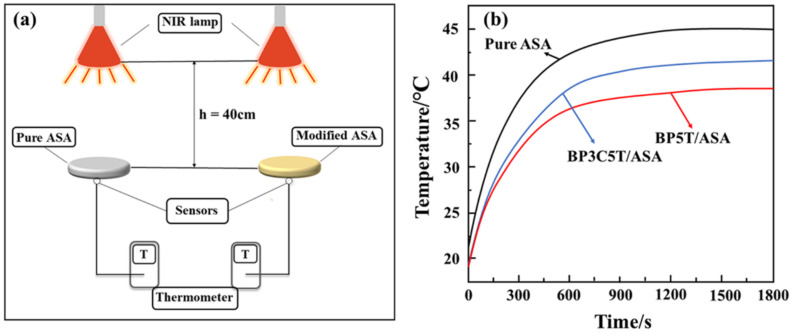
(**a**). NIR lamp radiation test setup, (**b**). Heating curves of pure ASA and modified ASA [4] Copyright 2021 with permission from Elsevier.

To improve ASA’s thermal insulation and weather resistance, chemicals have been added to ASA resin used as a construction material. The modified ASA has been subjected to an infrared radiation test, which revealed that the surface temperature of the specimen decreased by 6.4 °C. In addition, pigments with NIR reflectance enhanced the weather resistance of ASA. The setup for the radiation test to measure weather resistance with the help of a near infrared lamp is shown in Figure 1a, and the corresponding heat curves for pure and modified ASA are shown in Figure 1b [4].

**Figure 2:**
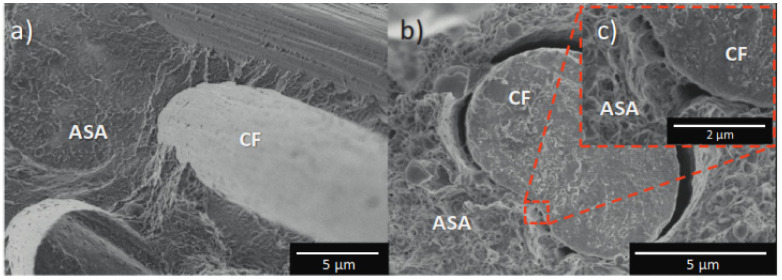
(**a**). SEM images showing flexural fracture at lower temperatures, (**b**). Tensile tested specimen, (**c**). Details of interface between carbon fiber and ASA [4] Copyright 2020 with permission from Elsevier.

The difference in interfacial tension between polymer pairs allows for the incorporation of two different elastomers, CPE and BR, into the core of ASA. Higher impact strengths were demonstrated by the mixes as a result of their testing [5]. After reinforcing ASA with carbon fiber and measuring its mechanical and thermal properties, the materials had superior tensile and flexural strength, and the thermal conductivity increased by 500 percent. Figure 2a depicts SEM images captured after a tensile test conducted at liquid nitrogen temperatures, whereas Figure 2b depicts the interaction between fiber and resin following flexural tests. The presence of a gap between the fiber and resin shows a lack of compatibility and interface strength, which results in fiber pullouts and degraded mechanical qualities [6].

**Figure 3:**
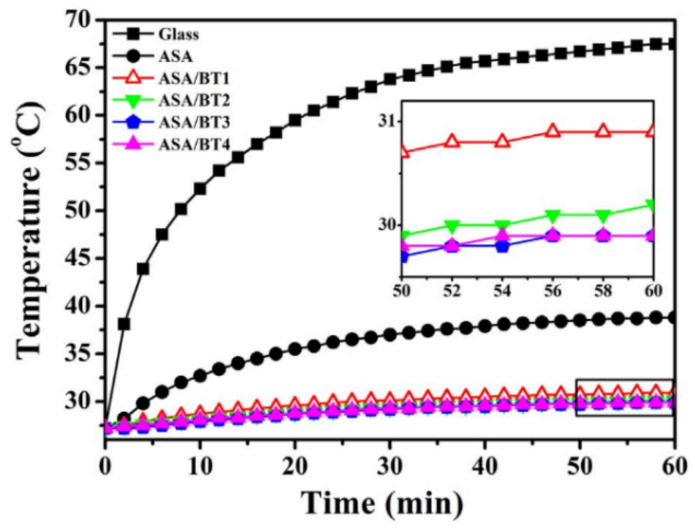
Inner temperature difference with respect to time [7] Copyright 2018 with permission from Elsevier.

ASA is combined with barium titanate and evaluated as a roof-cooling and solar-reflecting material. The introduction of barium titanate increased the NIR reflectance by two to three times, while the interior temperature dropped by 10 °C. Figure 3 depicts the increase in temperature of the specimen’s inner surface, which is used to measure solar reflectance. ASA with barium titanate (1068 nm) exhibited superior solar reflectance compared to other combinations, including glass [7].

**Figure 4:**
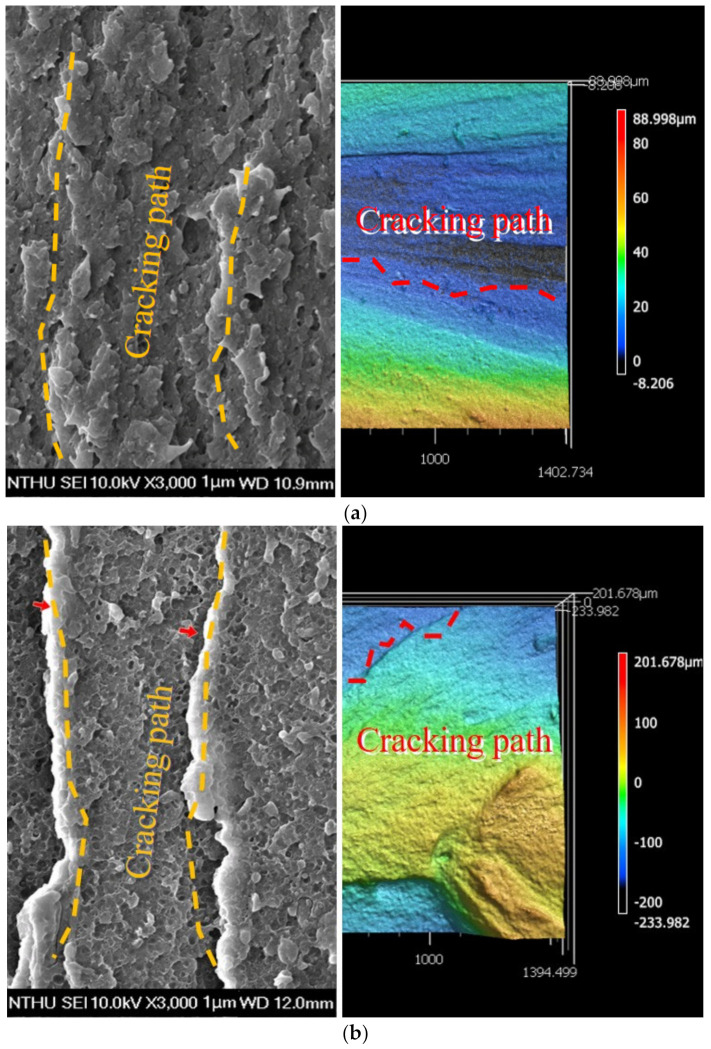
(**a**). Laser scanning confocal microscopy (Right). (**b**). SEM images of impact tested specimen showing the crack path [13] Reproduced with permission from MDPI.

The addition of antimony trioxide to ASA resin enhanced the cooling effect. Antimony trioxide exhibits solar-reflective qualities. When antimony trioxide is added to ASA, the water contact angle increases, indicating that the material can be used for roof cooling and composited as cool materials [8]. The use of 3D printing in the production of biological components is rising fast due to demand. PLA was discovered to be a biodegradable material suitable for use in the FDM production of bio-implants. The biodegradation test findings reveal good results throughout 30 to 60 days, and the weight loss was found to be satisfactory [9]. Stamp sand deposited as mine waste is combined with recycled ASA to create composites whose mechanical properties have been evaluated. It was noted that the tensile strength was reduced by half compared to recycled ASA in its pure form, and that stamp sand in conjunction with ABS produced positive results [10]. Metamaterials such as chiral, Re-entrant, and ASA are printed using FDM, and mechanical and ex situ tests revealed that structural alterations improved the materials’ properties, although inter-laminar fracture was seen during a compression test [11]. To determine the relationship between the morphologies of ASA composites and their mechanical properties, RAFT emulsion polymerization is used to generate block copolymers from ASA. By increasing the spherical particle size, the inter-particle distance can be decreased and the tensile characteristics can be improved. Additionally, the impact resistance was enhanced in comparison to triblock copolymer materials [12]. The technique of melt blending is used to create composites composed of polycarbonate, ASA and acrylic resin. It was noticed that the addition of acrylic resin boosted the impact strength and abrasion resistance. Additionally, it was discovered that the addition of acrylic resin reduced the tensile, hardness and melt flow rates marginally. The crack path in the composite was tracked with SEM and LSCM images as shown in Figure 4a,b [13].

Analyses of the effect of ASA addition to eucalyptus/PVC composites revealed that adding less than 15 percent ASA improves impact, tensile and flexural strengths. Adding 15 to 20 percent ASA to composites increased their thermal stability in the early stages but weakened them later due to ASA’s thermal degradation [14]. Thus, in this study, the ASA polymer is 3D printed utilizing the fused deposition modelling technique by altering five process parameters at three distinct levels while keeping other parameters constant. In earlier 3D printing research work, a maximum of three process parameters were taken into account and varied. In this work, however, a total of five process parameters were taken into consideration and their influence on mechanical properties was studied. The curiosity to understand the combined influence of the input factors motivated this work and makes it novel when compared with previous work carried out in this field. By evaluating the mechanical characteristics of the manufactured specimens, the influence of the parameters is examined and the results are optimized based on the analysis.

## 2. Materials and Manufacturing Process

### 2.1. Materials

River Polymer Industries of Gujarat, India, provided ASA polymer (acrylonitrile-co-styrene-co-acrylate). According to temperature tests, ASA’s strong solar reflectance prevents heat conduction and offers a more effective cooling effect in sunshine than glass. ASA features a high service temperature, low thermal conductivity and resistance to the elements. ASA has a density of 1.06–1.1 g/cm^3^ at 20 °C and a softening point of 85 to 100 °C, making it suitable for outdoor applications because it ignites at 400 °C. Low melting temperatures, which are 200–230 °C, 200–280 °C, and 200–250 °C, make it suitable for pipe extrusion, injection molding and thermoforming. Solvents including chloroform, dichlorobenzene, diethyl ether, ethyl benzoate, ethyl chloride, mesityl oxide, methyl chloride, methyl propyl ketone and xylene, dissolve ASA well. ASA is suited for outdoor sheet furniture, exterior cable enclosures and wide screen displays. ASA is a scratch-resistant polymer that can be utilized in automobile interiors and electrical devices. ASA has superior solar reflectivity and can be used in roof cooling materials [15]. The mechanical and physical properties of ASA are listed in Table 1.

### 2.2. Fabrication Method and Machinery Details

**Figure 5:**
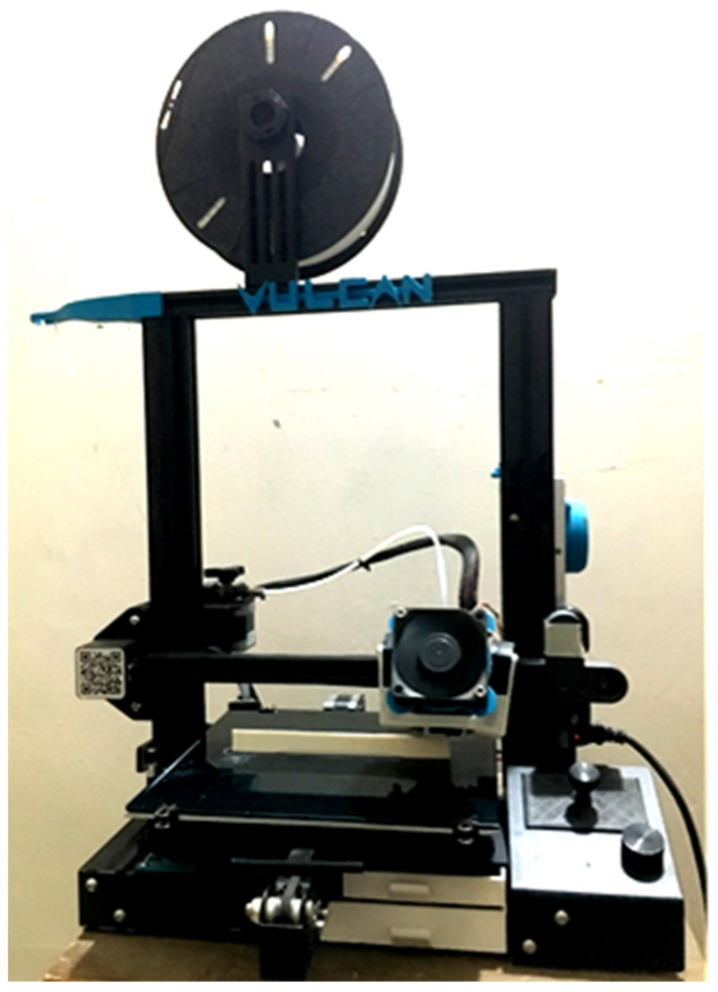
“Creality Ender—3” printer.

Using additive manufacturing, Acrylonitrile Styrene Acrylate (ASA) specimens for experimental work were printed on a “Creality Ender—3” FDM-based 3D printing system. The printer has a printing size of 220 × 220 × 250 mm and is based on the Cartesian system, with all three axes moving independently utilizing stepper motors. The ASA filament used in the experiment was a 1.75 mm-diameter wire that was kept on its mounting spool. All three samples were modelled in the CAD software Autodesk Fusion 360 and produced in the stl file format [16]. Then, the file was sliced in Ultimaker Cura slicing software according to the parameters, its G-code (machine code for the printer) was generated, and this was inserted into the 3D printer machine. Figure 5 depicts the 3D printer with which the specimens were printed.

### 2.3. Selected Process Parameters and Their Levels

The mechanical properties of components are greatly affected by printing parameters. Printing temperature, infill density, layer height, raster angle and printing orientation were chosen as experimental design factors to determine their effect on the specimen’s mechanical properties. The remaining printing parameters were adjusted to the same value for each sample. The printing temperatures for the setup were determined to be 245 °C, 255 °C and 265 °C. The infill density ranged from 30%, 70% and 100%. The height of the print layer ranged between 0.08 mm, 0.12 mm and 0.16 mm. The raster angle values for the experiment were 15°, 30° and 45°, and two print orientations, X-90 and Y-90, were obtained [17,18,19,20]. An experiment was designed by systematically altering each of these values, and samples were subjected to mechanical testing to determine the optimal printing conditions for achieving maximum strength. All other printing parameters, including printing speed of 60 mm/s, number of perimeters at 4, print bed temperature of 90 °C, 40 percent print cooling and the retraction settings, were held constant for all samples. The selected process parameters and their respective levels are listed in Table 2.

Using MINITAB, the experimental design for five components and three levels was prepared according to Taguchi’s experimental design. To avoid confusion and elicit precise results from a minimum number of trials, experimental designs were developed. By utilizing good experimental design, time, energy and money can be saved as fewer experiments will be required to attain the desired findings. The extra benefit of MINITAB software is that manual programming is not required, as the programs are already inbuilt into the software. A mixed-level design with two levels for the first factor (part orientation), and three levels for the remaining four factors, was selected in the software and an experimental design was created. The experimental design for the L18 orthogonal array is illustrated in Table 3. In accordance with the experimental design, the parameters were selected and the specimens were 3D-printed.

### 2.4. Mechanical Properties Evaluation Methods

**Figure 6:**
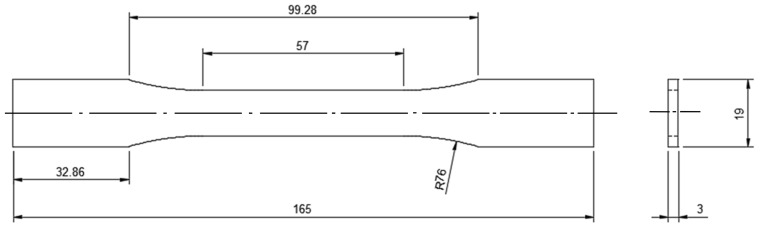
ASTM D638 specimen for tensile testing (all dimensions are in mm) [22].

**Figure 7:**
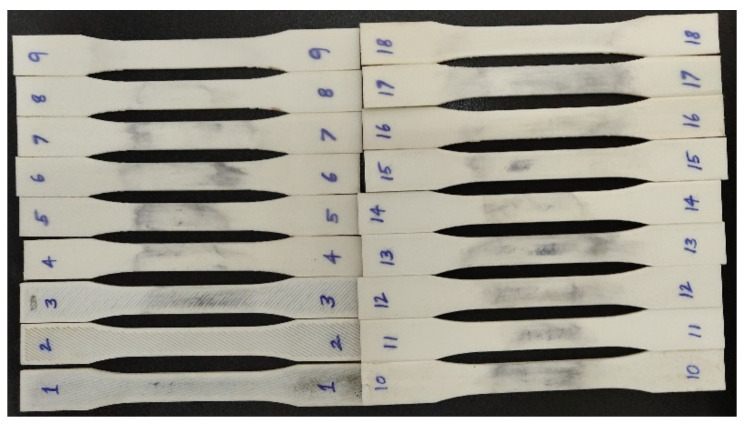
Printed specimens for tensile test as per the experimental design.

**Figure 8:**
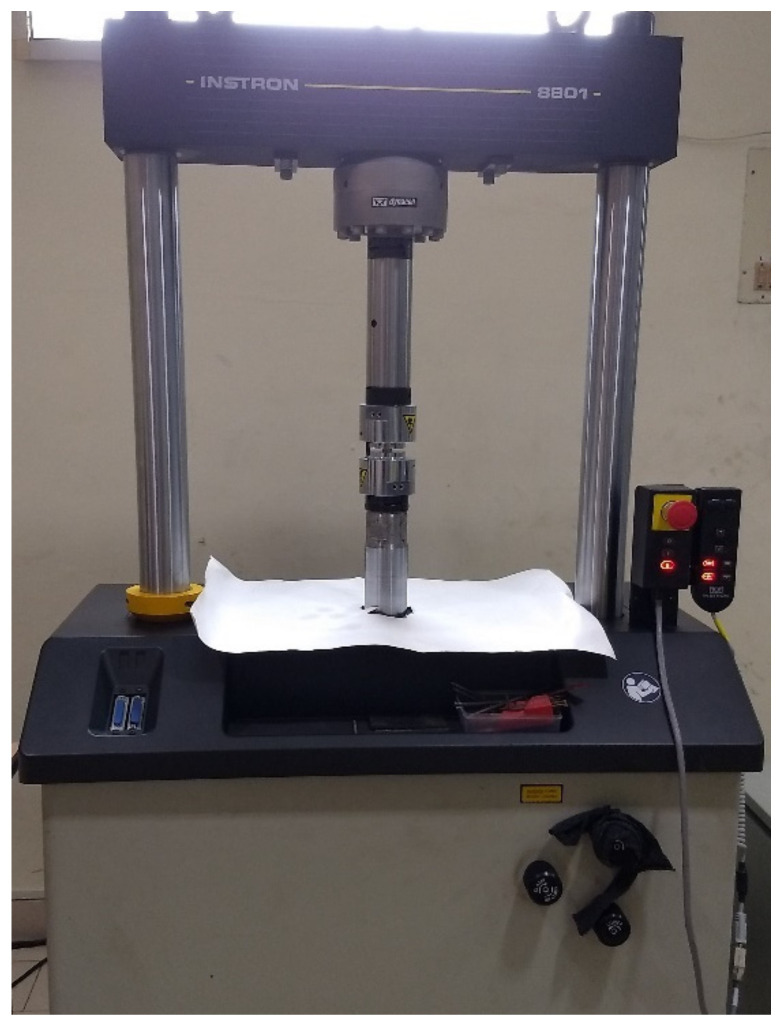
Instron 8801 UTM for tensile and bending test.

For evaluating the mechanical properties of 3D-printed ASA, the components for the tensile test, flexural test and impact test were selected in accordance with ASTM standards. For tensile testing, an ASTM D638 sample of 165 mm in length, 19 mm in width and 3 mm in thickness was selected. Figure 6 depicts the dumbbell-shaped specimen’s dimensions, whereas Figure 7 depicts the printed tensile test specimens. Figure 8 depicts an Instron 8801 UTM, the device utilized for tensile and flexural testing. The specimen was created in accordance with the experimental design by altering the process parameters. The printed samples were tidily packaged to prevent environmental contact and moisture contamination. The tensile test was conducted using an Instron 8801 model UTM machine with a 100 kN load capacity, two columns equipped with M30 load cells and actuators and a load capacity of 100 kN. The specimen was grasped in the grippers and testing commenced. During the test, the cross-head velocity and strain rate were both maintained at 5 mm/min. The tensile strength of the printed specimen can be found using Equation (1) [21].

Tensile strength (MPa) = Maximum load during breaking (Newton)/Cross-sectional area of the specimen (mm^2^) (1)

**Figure 9:**
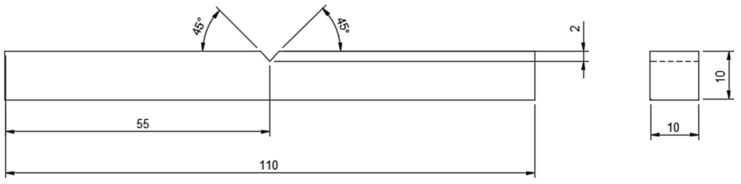
ASTM D6110 specimen for impact testing.

**Figure 10:**
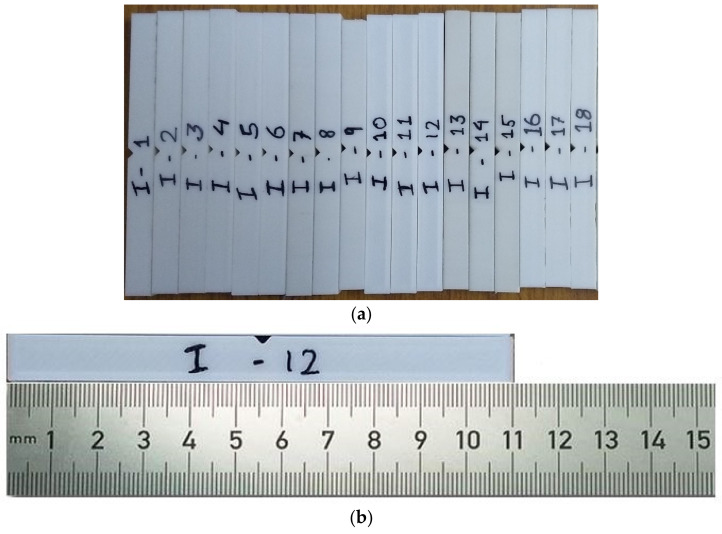
(**a**) Printed specimens for impact test as per the experimental design. (**b**). Impact specimen with scale.

**Figure 11:**
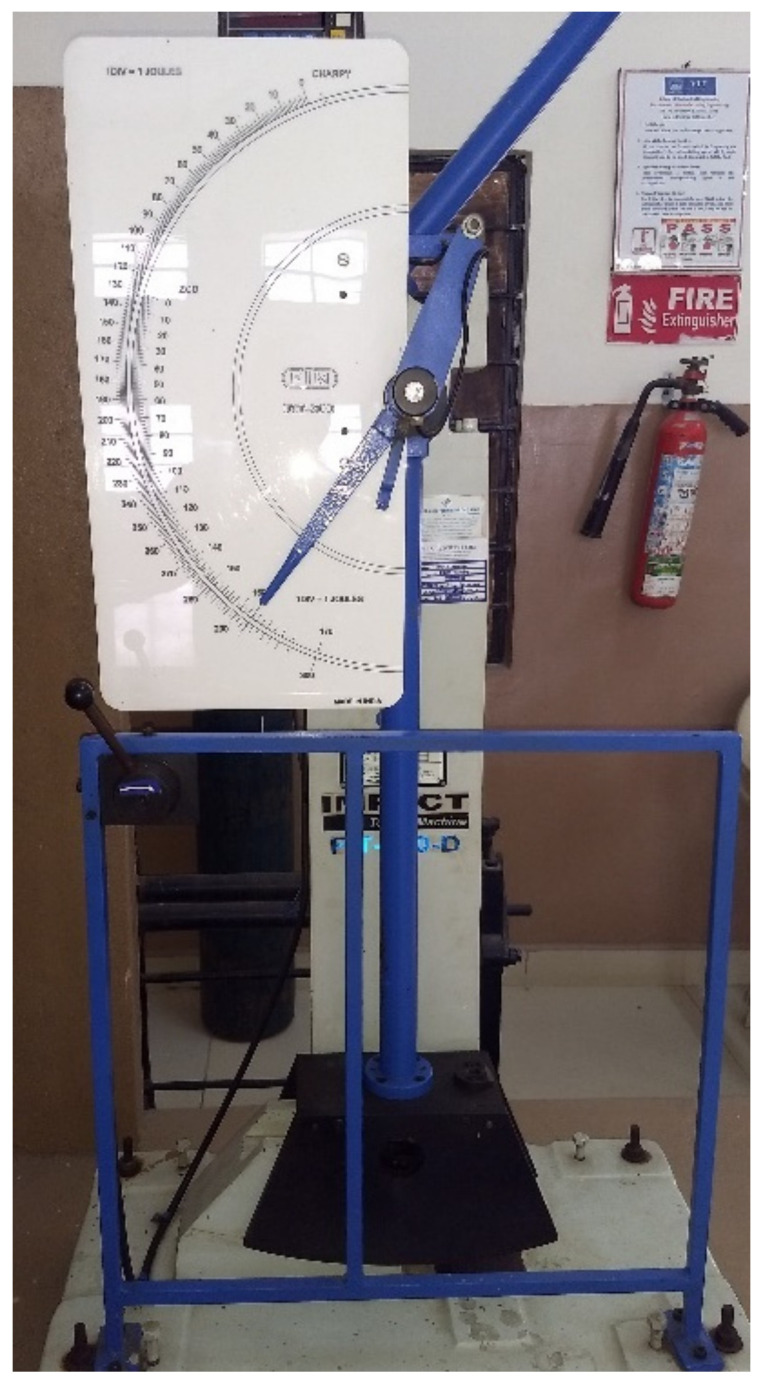
FIT-300-D model impact tester.

For impact testing, an ASTM D6110 sample with dimensions of 110 mm in length, 10 mm in width and thickness, and a 45° notch angle at the specimen’s midpoint was selected. The impact test was conducted using FIT-300-D model pendulum-type impact testing equipment with a capacity of 300 J and a minimum count of 0.1 J. Chennai’s Herenba Instruments and Engineers supplied and calibrated the equipment. The test was conducted at 37 °C and normal atmospheric pressure. Initially, the striker was released without the location of the specimen and its error was evaluated. Figure 9 depicts the specimen dimensions according to ASTM standards, and Figure 10a,b depict the printed specimens prior to the impact test. Figure 11 depicts the machine configuration, and the specimen was placed in the horizontal work holding fixture with the notch facing away from the striker. The striker was unleashed and caused to strike the specimen in its center. After breaking, the energy absorbed by the specimen was recorded and the specimen’s impact strength was calculated using Equation (2).

Impact strength (Charpy test) (J/mm^2^) = Energy absorbed (Joules)/Cross-sectional area (mm^2^) (2)

**Figure 12:**
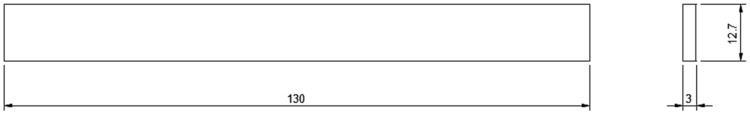
ASTM D790 specimen for flexural testing [23].

**Figure 13:**
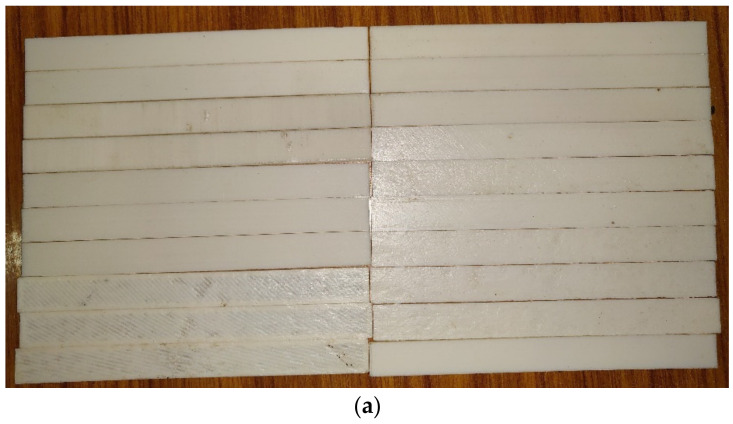

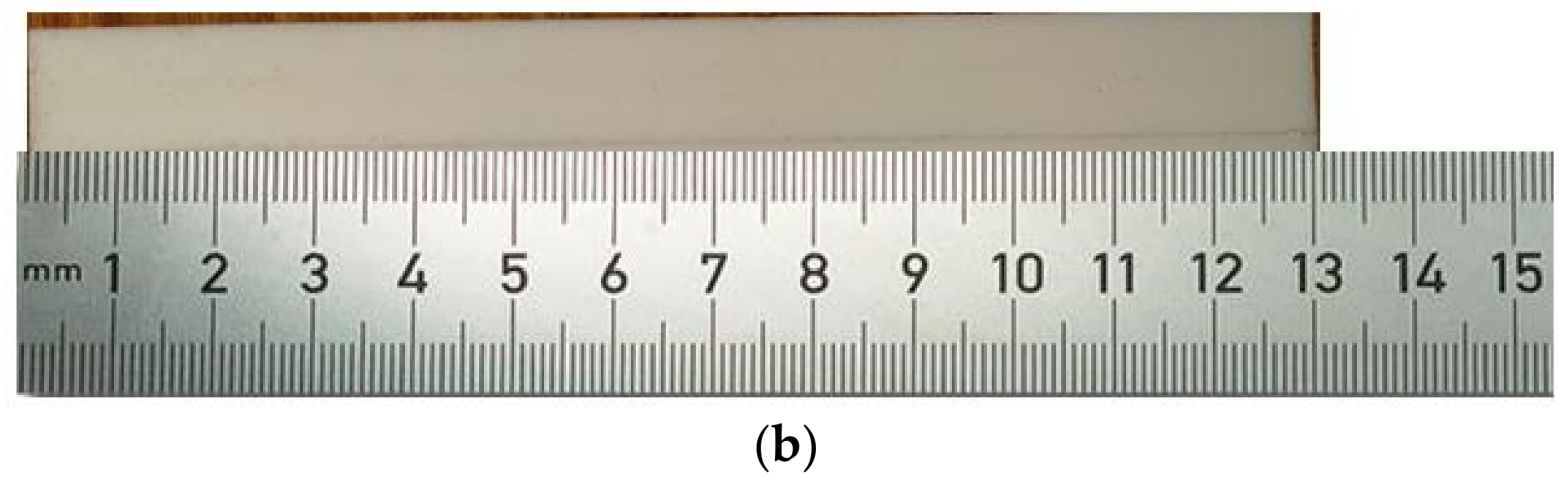
(**a**). Printed specimens for flexural test as per the experimental design. (**b**). Printed flexural specimen with scale.

For flexural testing, an ASTM D790 sample of 130 mm in length, 12.7 mm in width and 3 mm in thickness was chosen. The flexural test was conducted on the same Instron 8801 model UTM equipped with a unique attachment for three-point bending testing. Figure 12 depicts the dimensions of the specimen, whereas Figure 13a,b depict the printed specimens for the three-point bending test according to the experimental design. Using Equation (3), the specimen’s flexural strength was determined.

Flexural strength (MPa) = 3 × Load (N) × Span of support (mm)/2 × Specimen width (mm) × (specimen height)^2^ (mm) (3)

## 3. Results and Discussion

Table 4 contains the experimental design that varies five process parameters and the results of the tests that were conducted using that design. When compared to others, specimen 7 demonstrated greater values for all the test findings and had the greatest tensile strength rating, which was 50.57 MPa. It demonstrated the second greatest output in the flexural test, which shows that this particular design parametric combination produces superior mechanical qualities.

### 3.1. Tensile Strength

**Figure 14:**
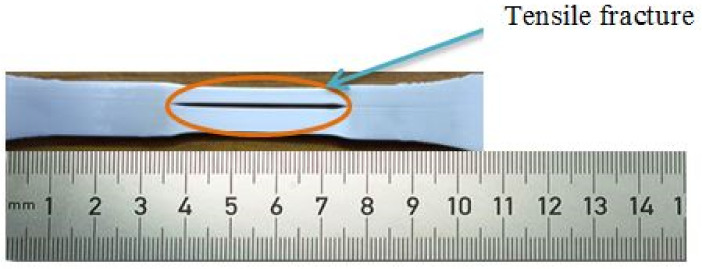
Tensile tested specimen.

The experimental data are provided as input to the MINITAB software for regression analysis in order to identify the most influential parameter which serves as the determining factor in producing output results. The tensile strength was determined to range between 39.97 MPa and 50.57 MPa. The majority of specimens subjected to tensile loads exhibited superior extension properties, as shown by the lengthening of the specimens prior to failure [24]. The majority of specimens exhibited plastic deformation prior to failure or cracking. The failure of the specimens happened approximately at the specimen’s center, indicating that the specimens are defect free and that the load acts at the proper place. It also demonstrated that the tests yield precise findings. The experimental values were then inputted into the software for regression analysis in order to determine the most influential parameter affecting the results. The coefficient of regression is 93.82%, which is closer to the acceptable value. The most influential criteria for tensile strength were found to be layer height and infill density. The specimens subjected to tensile testing demonstrated an extended fracture failure mechanism rather than a brittle fracture. Figure 14 depicts the deformed portion following a tensile test, which has an extended section due to plastic deformation. Due to enhanced tensile characteristics, the specimen did not fail completely while undergoing deformation. Table 5 represents the elongation of the specimen at break and yield point values. The ANOVA findings are presented in Table 6, where the Fisher value of each selected parameter reflects the extent of its influence.

**Figure 15:**
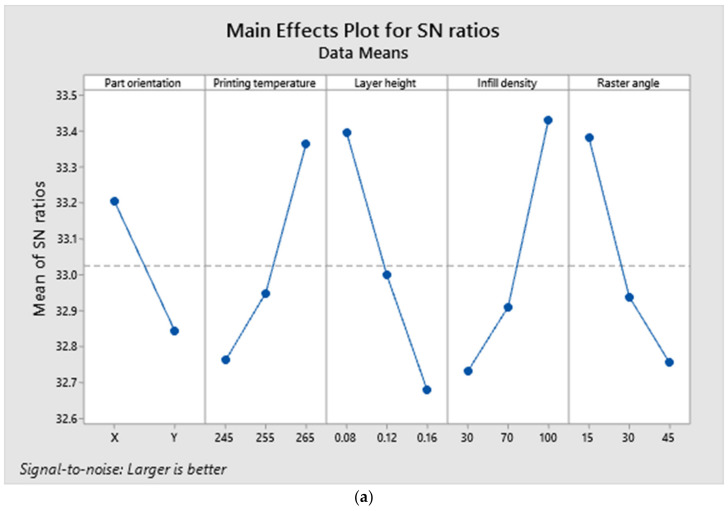

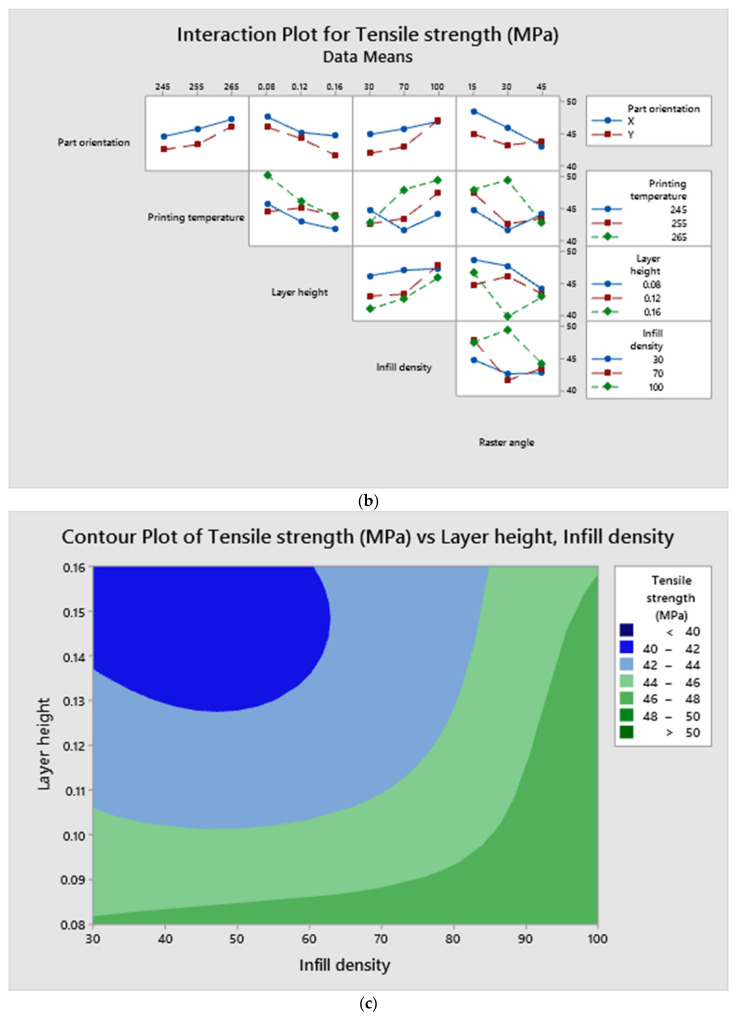

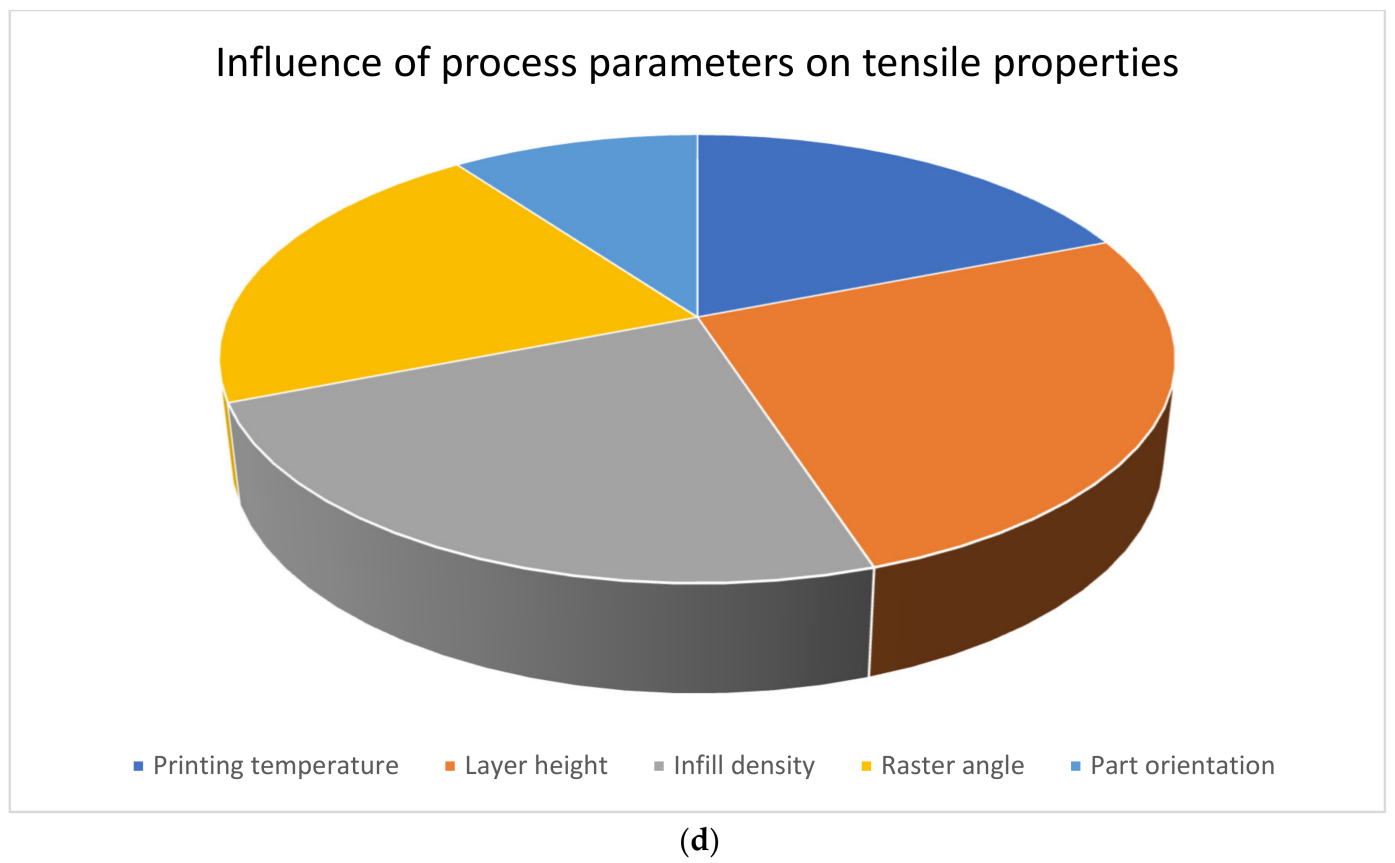
(**a**). Main effects plot for tensile test responses. (**b**). Interaction plot for tensile test responses. (**c**). Contour plot for maximum tensile strength. (**d**). Contribution plot of parameters for tensile strength.

Figure 15a–d depict the main effects, interaction, contour and contribution plots for tensile tested results. The larger is better concept was followed when plotting the S/N ratio’s main effects. It was projected that ‘X’ part orientation, printing temperature of 265 C, layer height of 0.08 mm, 100 percent infill density and a raster angle of 15 (A1, B3, C1, D3, E1) would result in the highest tensile strength. The interaction plot reveals that there is no interaction between printing temperature and part orientation, other than the fact that all components are dependent on one another. The contour plot was drawn between the layer height and the infill density, as these were the most influential parameters among the others. In order to achieve higher tensile strengths, the contour plot indicates that the layer height and infill density must be 0.08 mm and 100 percent, respectively. 

### 3.2. Flexural Strength

**Figure 16:**
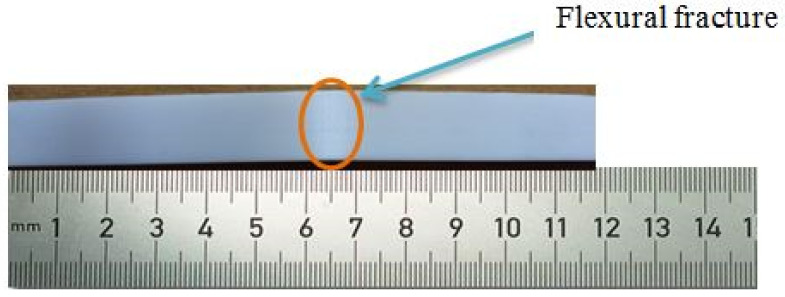
Deformed specimen after flexural test.

The specimen with the highest tensile strength also possessed superior flexural properties. Specimen number 16, having a ‘Y’ axis part orientation printed at 265 °C, with a layer height of 0.08 mm, a 100 percent infill density and a raster angle of 30 degrees, exhibited a maximum flexural strength of 82.87 MPa. Figure 16 depicts the specimen following the flexural test, which demonstrates that even after failure the cracks are not visible to the naked eye. Due to the formation of microscopic fractures that do not propagate further, the specimen did not fail completely or shatter into two pieces. It demonstrates the flexural stiffness of the specimen and the behavior of ASA prior to failure. The flexural strength ANOVA findings are presented in Table 7, and according to the Fisher value, infill density was the most influential parameter, followed by layer height. In addition, the regression coefficient was determined to be greater than 90 percent, demonstrating the significance of the model.

**Figure 17:**
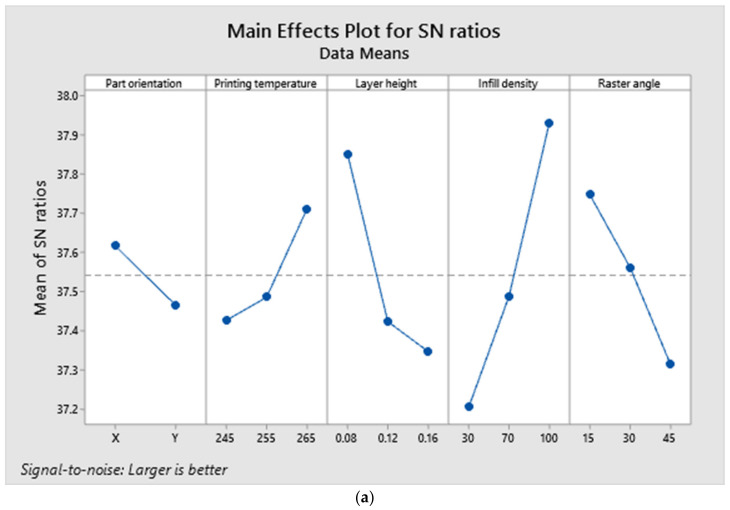

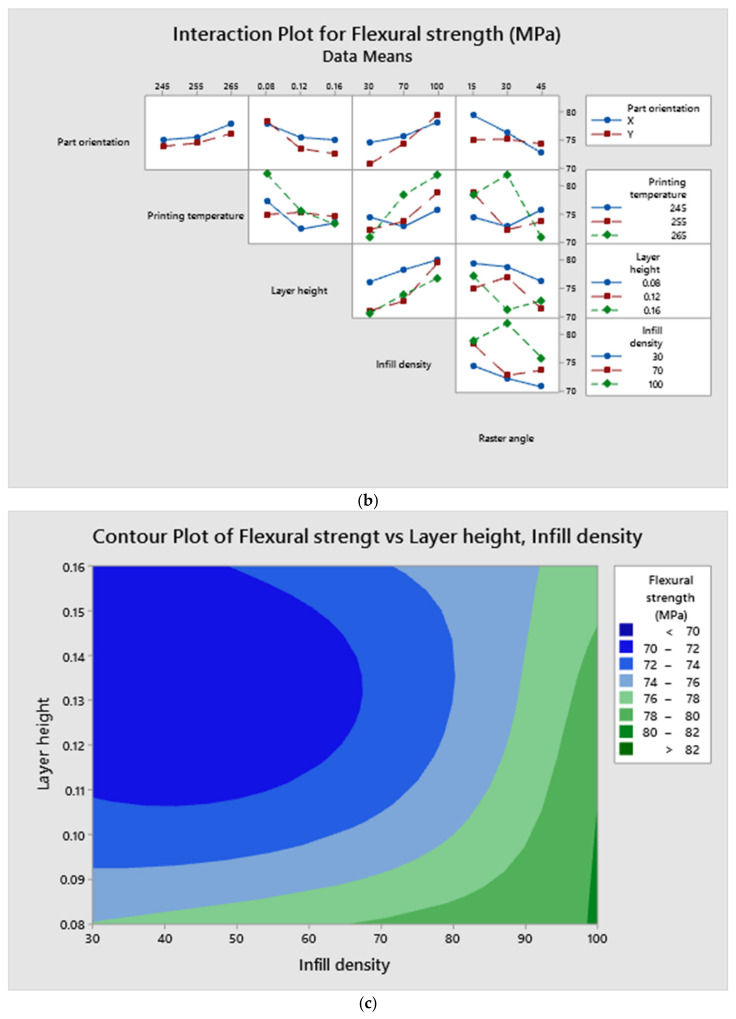

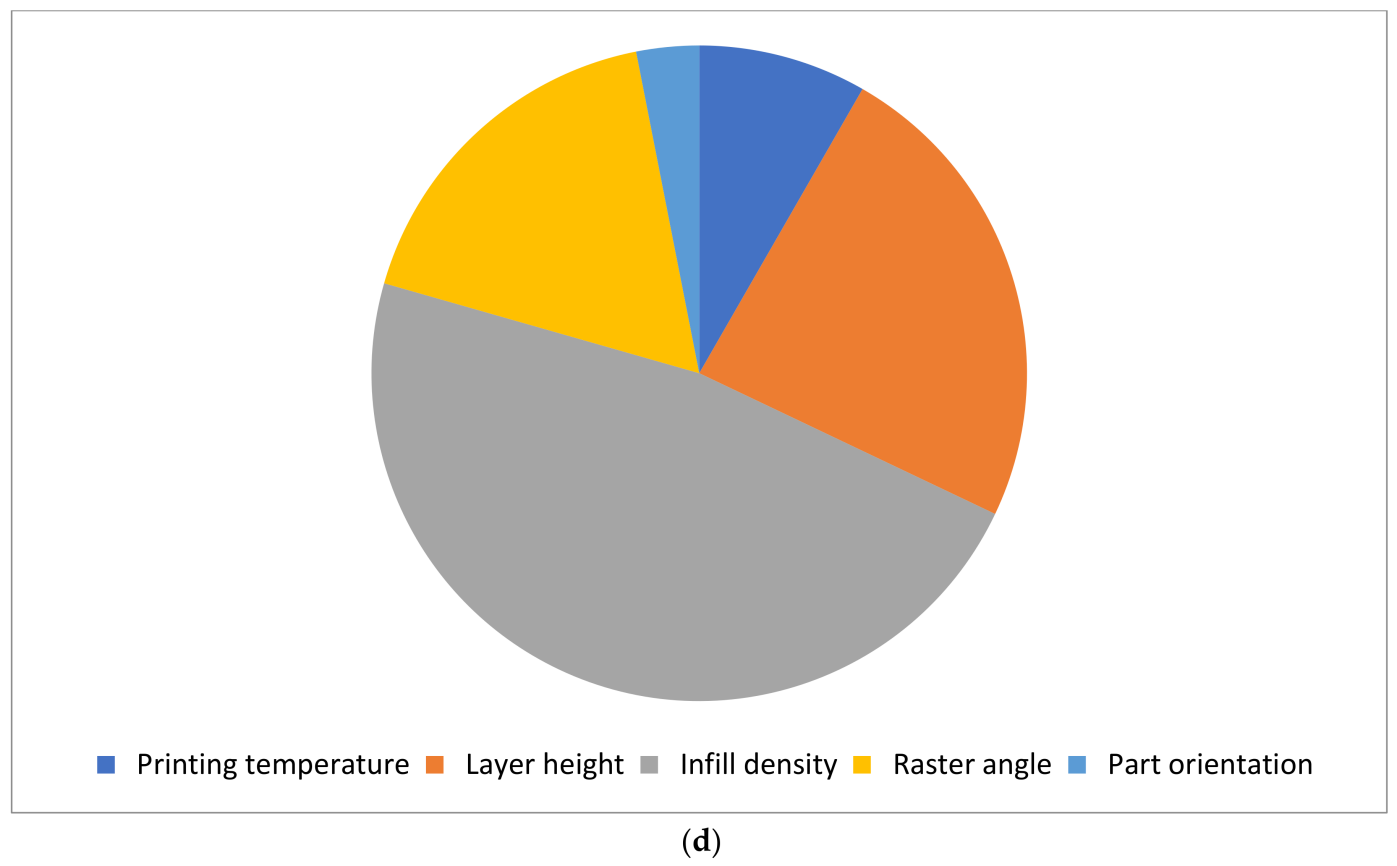
(**a**). Main effects plot for bending test responses. (**b**). Interaction plot for bending test responses. (**c**). Contour plot for maximum flexural strength. (**d**). Contribution of each factor for maximum flexural strength.

A1, B4, C1, D3 and E1 were the anticipated optimal values for maximal flexural strength, based on the main effects figure. The same predictions were made regarding the tensile property. It illustrates the relationship between tensile and flexural properties, which are directly proportional. The interaction plot provides the same information as tensile responses. The orientation of the part and the printing temperature are unrelated. Figure 17d depicts the effect of each parameter on the flexural properties as a percentage. Figure 17a–c depict the main effects, interaction and contour plots for the flexural strength results.

### 3.3. Impact Strength

**Figure 18:**
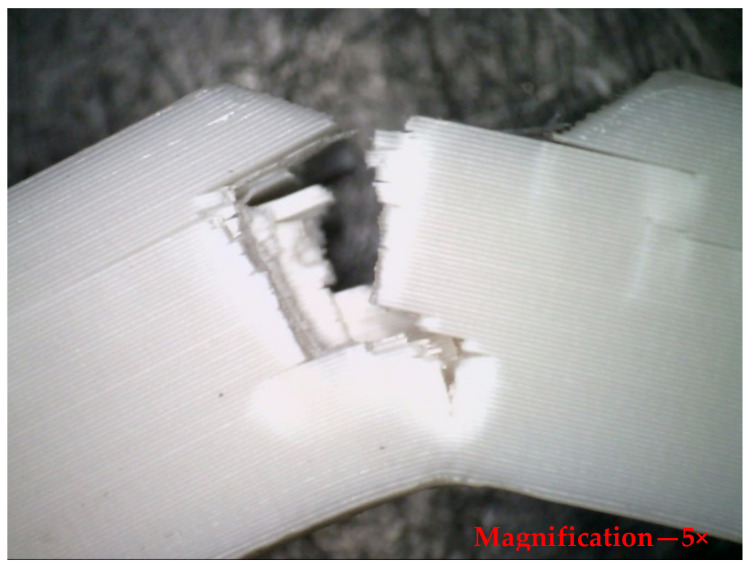
Optical microscopic view of the deformed specimen after impact test, side view.

The specimens with the highest impact resistance to sudden loads were numbers 7 and 16. The maximum impact strength observed was 0.17 J/mm^2^, whereas the minimum was 0.09 J/mm^2^. Figure 18 depicts the front view of a specimen that was fractured due to impact stresses with the effect of layer height and infill density evident. The crack began at the location of the notch and the layers impeded its progression. There are irregularities in the fracture propagation as the specimen was not shattered in a straight line. The specimen remained intact during the impact test, demonstrating its resistance to impact loads. The layer height appears to be the most influential factor in impact test analysis, as shown in Table 8 of the ANOVA results. With 93.01 percent, the regression coefficient is adequate.

**Figure 19:**
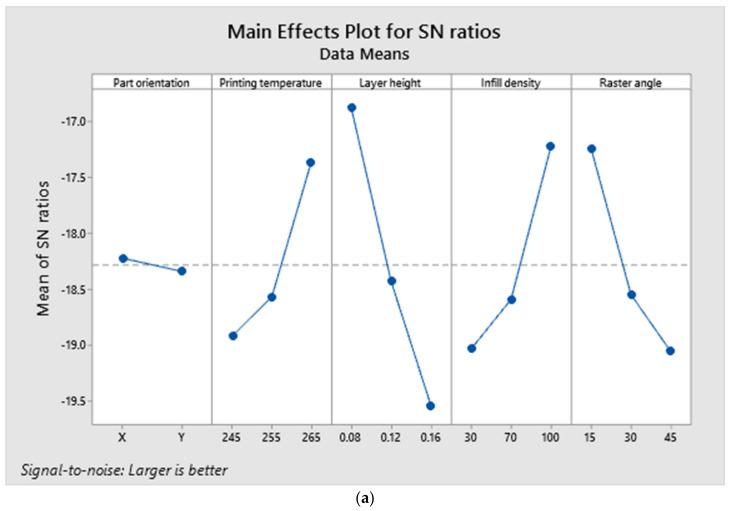

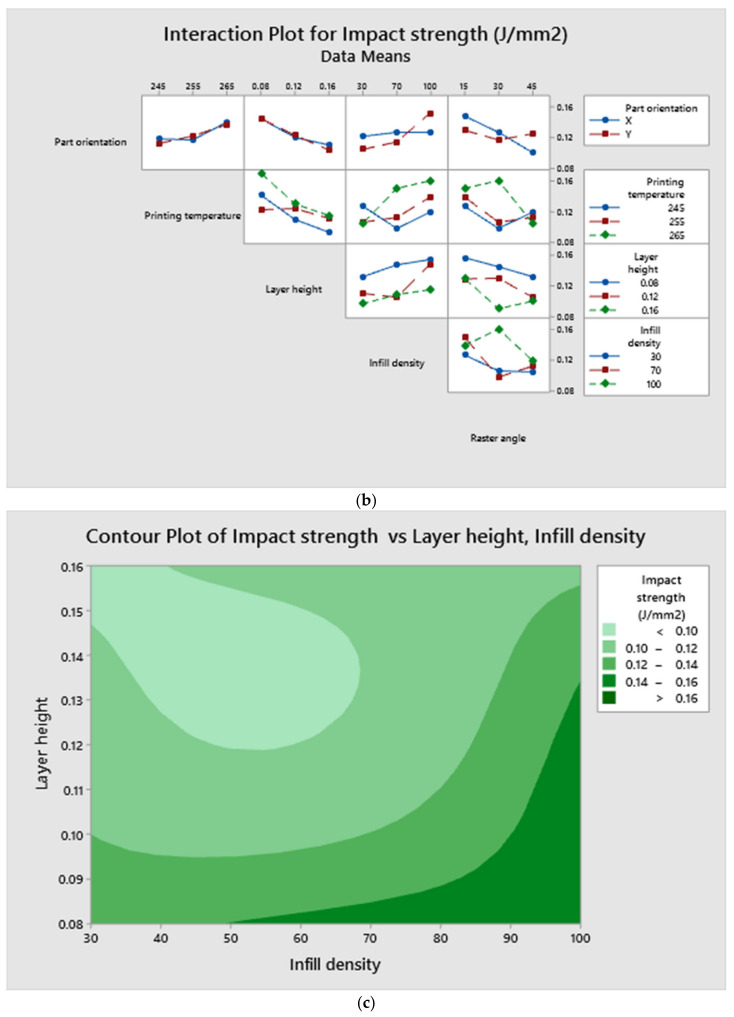

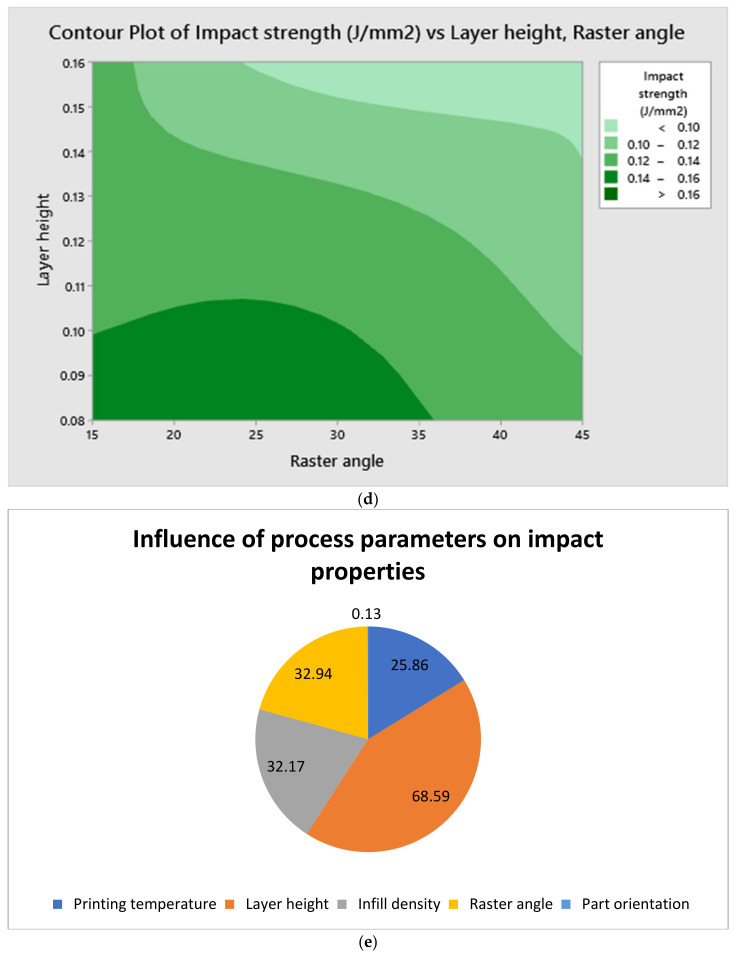
(**a**). Main effects plot for impact test responses. (**b**). Interaction plot for impact test responses. (**c**). Contour plot between layer height and infill density for maximum impact strength. (**d**). Contour plot between layer height and raster angle for maximum impact strength. (**e**). Contribution plot of various factors for maximum impact strength.

Figure 19a–d represent the main effects plot, the interaction plot and the contour plots, respectively. According to the forecast of the main effects plot, A1, B3, C1, D3 and E1 are the optimal values that result in greater impact strengths. It is apparent from the interaction plot that all the components are interrelated. Contour plots were created for infill density and layer height, as well as for layer height and raster angle, since these are the most influential elements in terms of impact strength [25]. The contribution and effect of each factor can be understood from Figure 19e.

## 4. Validation Test Details

**Figure 20:**
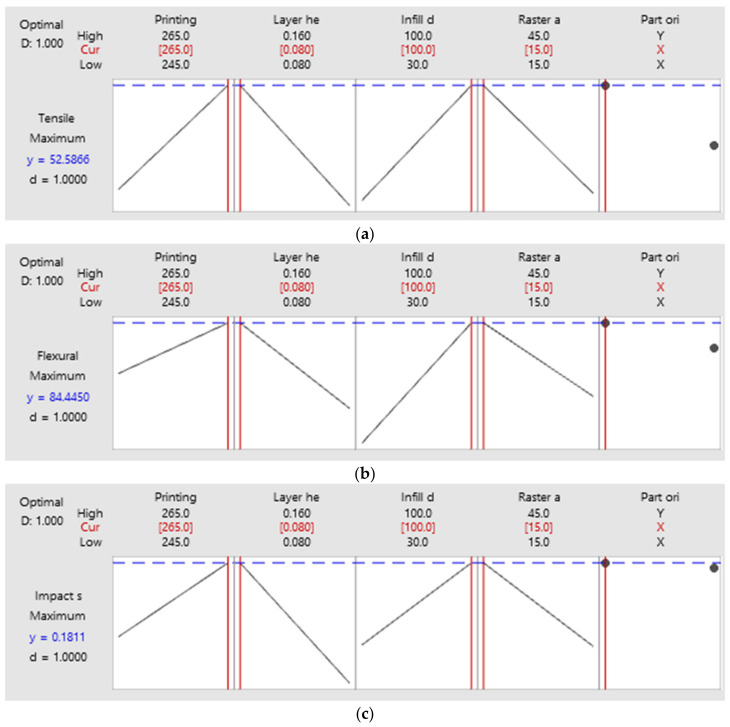
(**a**–**c**). Predicted results and optimum process parameters.

From the experimental data that were fed into the software, optimization outcomes and the factors that influence outputs were determined. The software predicted a result for each test and provided the optimal process parameters for the predicted result. Figure 20a–c depict the optimized parameters and the expected output for the optimal values. The comparison of anticipated and experimental values, together with the percentage of error, is displayed in Table 9. Specimens were prepared for each test using the optimum process parameters. For each test, three specimens were manufactured with identical process settings and their mean values were used to determine the final output. Validation was conducted by comparing the outcomes of each test, and the percentage of error was determined by computing the difference between the expected and experimental outcomes [26]. It was noticed that the error rate was less than 3 percent, which is within acceptable parameters.

## 5. Conclusions

- The L18 orthogonal array was constructed using the software MINITAB and specimens were produced in accordance with the experimental design.
- Specimens created with a 3D printer were tested to establish their mechanical properties in compliance with the standards and the results were then optimized.
- The optimized results enabled the identification of the process factors that had the largest impact on the output of each test, as well as the process parameters that produced the best results overall.
- The enhanced process parameters were then applied to the production of three further specimens, which were subsequently evaluated using the same criteria.
- The outcomes of the experiment were validated by comparing them to the predicted values based on the experiment’s results.
- When the specimen was built in accordance with the predicted input factors, tensile strength of 51.86 MPa, flexural strength of 82.56 MPa and impact strength of 0.180 J/mm^2^ were obtained.
- The proportion of inaccuracy discovered between the predicted and experimental results fell within the permitted range.
- A1B3C1D3E1 were the software’s projected input factors, and the software’s proposal was followed to make the specimens and validate the results.
- It was determined that the infill density and the layer height have the most effect on the outcomes when it comes to the fabrication of 3D-printed objects.

## 6. Future Scopes of Inquiry

**Figure 21:**
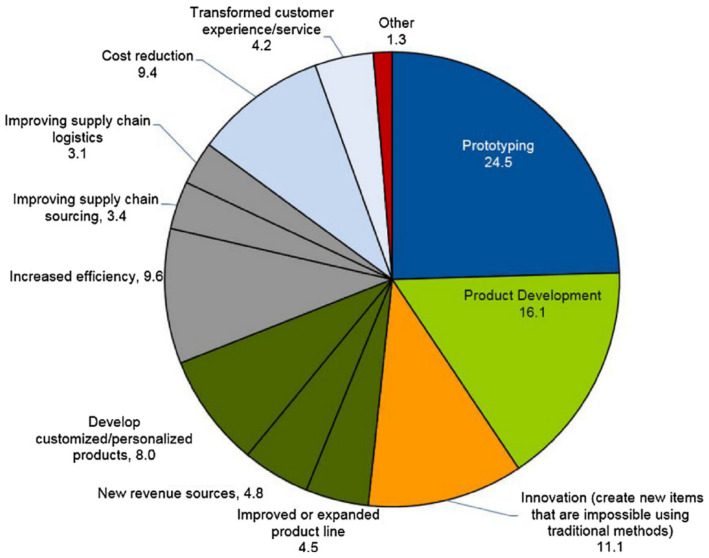
Contribution of 3D printing in various applications [27] Copyright 2018 with permission from Elsevier.

Already, elements and things that have been created using a 3D printer are being used in every industry. The advantages of 3D printing include the ease with which difficult profiles can be manufactured, less material waste, shortened production times and lower overall energy requirements. In the not-too-distant future, virtually all items and parts will be produced using 3D printing. A pie chart has been used in Figure 21 to show the many different applications for 3D printing technology. The quality of the items produced by additive manufacturing is also being improved through the utilization of a great number of recently developed methods and procedures. To further extend the scope of this work, the mechanical properties of the printed specimens could be improved by applying ceramic or metal coatings to them. The ASA polymer can be mixed with several fillers, each of which has the potential to contribute to an improvement in the polymer’s fundamental properties. Wear, water absorption, abrasion resistance, conductivity and flammability are some additional tests that can be carried out in addition to the ones already mentioned. It is possible to reinforce ASA with high performance fibers in order to determine the extent of the improvement in all qualities necessary for specific applications.

## Figures and Tables

**Table 1:** Properties of ASA [6]. Copyright 2020 with permission from Elsevier.

Properties	Units	Neat ASA
Ultimate tensile strength	MPa	45.7 ± 0.9
Ultimate flexural strength	MPa	52.6 ± 1.9
Impact strength	kJ/m^2^	140 ± 30
Material flow rate	g/10 min	47
Glass transition temperature	°C	103
Thermal conductivity	W/mK	0.17

**Table 2:** Process variables and their various stages.

S. No.	Variables	Units	Stages		
			1	2	3
1	Part orientation	axis	X	Y	-
2	Printing temperature	°C	245	255	265
3	Layer height	mm	0.08	0.12	0.16
4	Infill density	%	30	70	100
5	Raster angle	°	15	30	45

**Table 3:** L18 orthogonal array experimental design.

Trial No.	Process Parameters				
	Part Orientation (Axis)	Printing Temperature (°C)	Layer Height (mm)	Infill Density (%)	Raster Angle (°)
1	X	245	0.08	30	15
2	X	245	0.12	70	30
3	X	245	0.16	100	45
4	X	255	0.08	30	30
5	X	255	0.12	70	45
6	X	255	0.16	100	15
7	X	265	0.08	70	15
8	X	265	0.12	100	30
9	X	265	0.16	30	45
10	Y	245	0.08	100	45
11	Y	245	0.12	30	15
12	Y	245	0.16	70	30
13	Y	255	0.08	70	45
14	Y	255	0.12	100	15
15	Y	255	0.16	30	30
16	Y	265	0.08	100	30
17	Y	265	0.12	30	45
18	Y	265	0.16	70	15

**Table 4:** Experimental design with output responses.

Trial No.	Process Parameters					Responses		
	Part Orientation (Axis) (A)	Printing Temperature (°C) (B)	Layer Height (mm) (C)	Infill Density (%) (D)	Raster Angle (°) (E)	Tensile Strength (MPa)	Flexural Strength (MPa)	Impact Strength (J/mm^2^)
1	X	245	0.08	30	15	46.84	77.45	0.14
2	X	245	0.12	70	30	43.20	73.11	0.11
3	X	245	0.16	100	45	43.54	74.35	0.10
4	X	255	0.08	30	30	45.50	74.74	0.12
5	X	255	0.12	70	45	43.41	72.28	0.10
6	X	255	0.16	100	15	48.19	79.34	0.13
7	X	265	0.08	70	15	50.57	81.43	0.17
8	X	265	0.12	100	30	48.95	80.90	0.15
9	X	265	0.16	30	45	42.19	71.23	0.10
10	Y	245	0.08	100	45	44.72	77.32	0.14
11	Y	245	0.12	30	15	42.76	71.60	0.11
12	Y	245	0.16	70	30	39.97	72.53	0.09
13	Y	255	0.08	70	45	43.46	75.12	0.13
14	Y	255	0.12	100	15	46.72	78.32	0.15
15	Y	255	0.16	30	30	39.67	69.82	0.09
16	Y	265	0.08	100	30	49.88	82.87	0.17
17	Y	265	0.12	30	45	43.26	70.36	0.11
18	Y	265	0.16	70	15	45.32	75.21	0.13

**Table 5:** Elongation at break and yield point values for tensile test results.

Trial No.	Responses		
	Tensile Strength (MPa)	Elongation at Break (mm)	Yield Point (MPa)
1	46.84	25	52.32
2	43.20	12	48.87
3	43.54	9	45.35
4	45.50	14	51.67
5	43.41	7	47.75
6	48.19	24	53.83
7	50.57	34	59.13
8	48.95	21	56.82
9	42.19	15	47.26
10	44.72	18	50.1
11	42.76	26	47.98
12	39.97	10	45.73
13	43.46	16	50.9
14	46.72	28	53.42
15	39.67	7	46.82
16	49.88	29	57.12
17	43.26	15	50.13
18	45.32	20	52.47

**Table 6:** Tensile test response ANOVA.

Source	DF	Adj SS	Adj MS	F-Value	*p*-Value
Regression	5	156.8	31.3595	36.44	0
Printing temperature	1	30.53	30.5283	35.47	0
Layer height	1	40.66	40.664	47.25	0
Infill density	1	37.51	37.505	43.58	0
Raster angle	1	32.74	32.736	38.04	0
Part orientation	1	15.36	15.3643	17.85	0.001
Error	12	10.33	0.8606		
Total	17	167.12			
S	R-sq		R-sq (adj)	R-sq (pred)	
0.92769	93.82%		91.25%	85.64%	

**Table 7:** Flexural test response ANOVA.

Source	DF	Adj SS	Adj MS	F-Value	*p*-Value
Regression	5	245.4	49.08	26.59	0
Printing temperature	1	20.384	20.384	11.04	0.006
Layer height	1	58.314	58.314	31.6	0
Infill density	1	116.216	116.216	62.97	0
Raster angle	1	42.903	42.903	23.25	0
Part orientation	1	7.583	7.583	4.11	0.065
Error	12	22.147	1.846		
Total	17	267.547			
S	R-sq		R-sq (adj)	R-sq (pred)	
1.35853	91.72%		88.27%	81.70%	

**Table 8:** Impact test response ANOVA.

Scheme	DF	Adj SS	Adj MS	F-Value	*p*-Value
Regression	5	0.010085	0.002017	31.94	0
Printing temperature	1	0.001633	0.001633	25.86	0
Layer height	1	0.004332	0.004332	68.59	0
Infill density	1	0.002032	0.002032	32.17	0
Raster angle	1	0.00208	0.00208	32.94	0
Part orientation	1	0.000008	0.000008	0.13	0.728
Error	12	0.000758	0.000063		
Total	17	0.010843			
S	R-sq		R-sq (adj)	R-sq (pred)	
0.007947	93.01%		90.10%	82.68%	

**Table 9:** Predicted and experimental results with error percentage.

Output Factors	Optimal Control Parameters		Error (%)
	Predicted	Experimental	
Tensile strength in MPa	A1B3C1D3E1	A1B3C1D3E1	1.31
	52.58	51.89	
Flexural strength in MPa	A1B3C1D3E1	A1B3C1D3E1	2.22
	84.44	82.56	
Impact strength in J/mm^2^	A1B3C1D3E1	A1B3C1D3E1	0.55
	0.181	0.180	

## Data Availability

Not applicable.

## Nomenclature

ASA	Acrylonitrile styrene acrylate
NIR	Near infrared radiation
CPE	Chlorinated Polyethylene
BR	Polybutadiene rubber
3D	Three dimensional
FDM	Fused deposition modelling
ASTM	American Society for Testing and Materials
SEM	Scanning electron microscopy
RAFT	Reversible addition–fragmentation chain-transfer
LSCM	Laser scanning confocal microscopy
PVC	Polyvinyl chloride
ANOVA	Analysis of variance
PLA	Polylactic acid
ABS	Acrylonitrile butadiene styrene

## References

[1] Jakus A.E. (2018). An Introduction to 3D Printing-Past, Present, and Future Promise. 3D Print. Orthop. Surg..

[2] Delda R.N.M., Basuel R.B., Hacla R.P., Martinez D.W.C., Cabibihan J.-J., Dizon J.R.C. (2021). 3D Printing Polymeric Materials for Robots with Embedded Systems. Technologies.

[3] Thiam B.G., El Magri A., Vanaei H.R., Vaudreuil S. (2022). 3D Printed and Conventional Membranes—A Review. Polymers.

[4] Tian M., Chen C., Han A., Ye M., Chen X. (2021). Estimating Thermal Insulation Performance and Weather Resistance of Acrylonitrile-Styrene-Acrylate Modified with High Solar Reflective Pigments: Pr^3+^/Cr^3+^ Doped BaTiO_3_. Sol. Energy.

[5] Mao Z., Zhang J. (2018). Largely Improved the Low Temperature Toughness of Acrylonitrile-Styrene-Acrylate (ASA) Resin: Fabricated a Core-Shell Structure of Two Elastomers through the Differences of Interfacial Tensions. Appl. Surf. Sci..

[6] Sánchez D.M., de la Mata M., Delgado F.J., Casal V., Molina S.I. (2020). Development of Carbon Fiber Acrylonitrile Styrene Acrylate Composite for Large Format Additive Manufacturing. Mater. Des..

[7] Xiang B., Zhang J. (2018). A New Member of Solar Heat-Reflective Pigments: BaTiO3 and Its Effect on the Cooling Properties of ASA (Acrylonitrile-Styrene-Acrylate Copolymer). Sol. Energy Mater. Sol. Cells.

[8] Qi Y., Zhang J. (2019). Chemically Modified Sb_2_O_3_, a New Member of High Solar-Reflective Material Family, Incorporating with ASA (Acrylonitrile-Styrene-Acrylate Copolymer) for Fabrication of Cooling Composite with Lower Wetting Behavior. Compos. Part B Eng..

[9] Raj S.A., Muthukumaran E., Jayakrishna K. (2018). A Case Study of 3D Printed PLA and Its Mechanical Properties. Mater. Today Proc..

[10] Meyer T.K., Tanikella N.G., Reich M.J., Pearce J.M. (2020). Potential of Distributed Recycling from Hybrid Manufacturing of 3-D Printing and Injection Molding of Stamp Sand and Acrylonitrile Styrene Acrylate Waste Composite. Sustain. Mater. Technol..

[11] Magadum S., Gilorkar A., Amol Deepak M., Rakshith B.S. (2021). Design, Simulation and Experimental Investigation of 3D Printed Mechanical Metamaterials. Proceedings of the 2021 International Solid Freeform Fabrication Symposium 2021.

[12] Huang J., Luo Y., Gao X. (2019). Morphology and Mechanical Properties of Acrylonitrile-Styrene-Acrylate Toughened Plastics with Block Copolymer Chain Structure. Polym. Eng. Sci..

[13] Huang J., Kuo C., Tsai H. (2022). Stiffness Enhancement, Anti-Aging, and Self-Forming Holes in Polycarbonate/Acrylonitrile-Styrene-Acrylic by the Core-Shell Structure of Acrylic ResinStructure of Acrylic Resin. Polymers.

[14] Zhang K., Hamza Bichi A., Yang J. (2021). Effect of Acrylonitrile Styrene Acrylate on Mechanical, Thermal and Three-Body Abrasion Behaviors of Eucalyptus Fiber Reinforced Polyvinyl Chloride Composite. Mater. Res. Express.

[15] Xiang B., Zhang J. (2018). Effects of Content and Surface Hydrophobic Modification of BaTiO 3 on the Cooling Properties of ASA (Acrylonitrile-Styrene-Acrylate Copolymer). Appl. Surf. Sci..

[16] Veselý P. Nozzle Temperature Effect on 3D Printed Structure Properties. Proceedings of the ELEKTROTECHNOLÓGIA 2019.

[17] Dey A., Yodo N. (2019). A Systematic Survey of FDM Process Parameter Optimization and Their Influence on Part Characteristics. J. Manuf. Mater. Process..

[18] Ramezani Dana H., Barbe F., Delbreilh L., Ben Azzouna M., Guillet A., Breteau T. (2019). Polymer Additive Manufacturing of ABS Structure: Influence of Printing Direction on Mechanical Properties. J. Manuf. Process..

[19] Popescu D., Zapciu A., Amza C., Baciu F., Marinescu R. (2018). FDM Process Parameters Influence over the Mechanical Properties of Polymer Specimens: A Review. Polym. Test..

[20] Raam Kumar S., Sridhar S., Venkatraman R., Venkatesan M. (2020). Polymer Additive Manufacturing of ASA Structure: Influence of Printing Parameters on Mechanical Properties. Mater. Today Proc..

[21] Ganapathy S.B., Sakthivel A.R., Sultan M.T.H., Shahar F.S., Shah A.U.M., Khan T., Sebaey T.A. (2022). Effect of Prosopis Juliflora Thorns on Mechanical Properties of Plastic Waste Reinforced Epoxy Composites. Polymers.

[22] (2014). Standard Test Method for Tensile Properties of Plastics.

[23] (2017). Standard Test Methods for Flexural Properties of Unreinforced and Reinforced Plastics and Electrical Insulating Materials.

[24] Guessasma S., Belhabib S., Nouri H. (2019). Microstructure, Thermal and Mechanical Behavior of 3D Printed Acrylonitrile Styrene Acrylate. Macromol. Mater. Eng..

[25] Ezeh O.H., Susmel L. (2019). Fatigue Strength of Additively Manufactured Polylactide (PLA): Effect of Raster Angle and Non-Zero Mean Stresses. Int. J. Fatigue.

[26] Sakthi Balan G., Nandha Gopan S., Santhosh Kumar V., Ravichandran M. (2020). Effect of Chemical Treatment on Mechanical Properties of Prawn Antenna Reinforced Waste Plastic Particulates Filled Polymer Composites. Mater. Today Proc..

[27] Dizon J.R.C., Espera A.H., Chen Q., Advincula R.C. (2018). Mechanical Characterization of 3D-Printed Polymers. Addit. Manuf..

